# How Is RAN Related to Reading Fluency? A Comprehensive Examination of the Prominent Theoretical Accounts

**DOI:** 10.3389/fpsyg.2016.01217

**Published:** 2016-08-24

**Authors:** Timothy C. Papadopoulos, George C. Spanoudis, George K. Georgiou

**Affiliations:** ^1^Psychology, Center for Applied Neuroscience, University of CyprusNicosia, Cyprus; ^2^Educational Psychology, J. P. DAS Developmental Disabilities Centre, University of AlbertaEdmonton, AB, Canada

**Keywords:** rapid automatized naming, reading fluency, longitudinal, Greek

## Abstract

We examined the prominent theoretical explanations of the RAN-reading relationship in a relatively transparent language (Greek) in a sample of children (*n* = 286) followed from Grade 1 to Grade 2. Specifically, we tested the fit of eight different models, as defined by the type of reading performance predicted (oral vs. silent word reading fluency), the type of RAN tasks (non-alphanumeric vs. alphanumeric), and the RAN effects (direct vs. indirect). Working memory, attention, processing speed, and motor skills were used as “common cause” variables predicting both RAN and reading fluency and phonological awareness and orthographic processing were used as mediators of RAN's effects on reading fluency. The findings of both concurrent and longitudinal analyses indicated that RAN is a unique predictor of oral reading fluency, but not silent reading fluency. Using alphanumeric or non-alphanumeric RAN did not particularly affect the RAN-reading relationship. Both phonological awareness and orthographic processing partly mediated RAN's effects on reading fluency. Theoretical implications of these findings are discussed.

## Introduction

Several studies have established that rapid automatized naming (RAN), defined as the ability to name as fast as possible visually presented stimuli such as colors, objects, digits, and letters, is a strong predictor of reading in every language studied thus far (e.g., de Jong and van der Leij, 1999; Compton, 2003; Parrila et al., 2004; Cho and McBride-Chang, 2005; Lepola et al., 2005; Savage and Frederickson, 2005; Georgiou et al., 2008a; Landerl and Wimmer, 2008; Vaessen and Blomert, 2010; Ziegler et al., 2010; Taibah and Haynes, 2011; Nag and Snowling, 2012; Moll et al., 2014). In a recent meta-analysis, the size of their relationship has been estimated to be 0.48 (Araújo et al., 2015). Despite the acknowledged importance of RAN in predicting reading, the reason why RAN predicts reading is still a matter of debate (e.g., Kirby et al., 2010, for a review). Wolf et al. (2000) suggested that this uncertainty emanates from RAN's multi-componential nature since it requires the coordination of several sub-processes such as attentional, phonological, orthographic, memory, motoric, and articulatory processes, and anyone of these could drive its relationship with reading. Unfortunately, to date, no studies have examined the joint and unique contribution of these sub-processes in the RAN-reading relationship. Thus, the purpose of this study was to examine which one of these sub-processes or a combination of them is responsible for the relationship between RAN and word reading by simultaneously contrasting their role in the RAN-reading relationship.

During the last two decades several theoretical accounts have been proposed to explain the RAN-reading relationship. Initially, Torgesen, Wagner, and their colleagues proposed that RAN is related to reading because they both require efficient access to, and retrieval of, phonological representations from long-term memory (e.g., Wagner and Torgesen, 1987; Torgesen et al., 1994, 1997). This theoretical account is still popular (e.g., Bowey et al., 2005; Savage et al., 2007; Ziegler et al., 2010). However, several pieces of evidence challenge its validity. First, RAN has been shown to account for variance in reading over and above the effects of other measures of phonological processing such as phonological awareness (e.g., de Jong and van der Leij, 1999; Parrila et al., 2004; Vaessen and Blomert, 2010) and phonological short-term memory (e.g., Bowers et al., 1988; Parrila et al., 2004; Georgiou et al., 2008a). Second, children with both phonological awareness and RAN deficits have been found to experience more severe reading difficulties compared to children with deficits in either RAN or phonological awareness (e.g., Manis et al., 2000; Kirby et al., 2003; Papadopoulos et al., 2009a). Third, phonological awareness and RAN appear to predict different kinds of reading outcomes. RAN is a stronger predictor of reading fluency and phonological awareness is a stronger predictor of reading accuracy (e.g., Savage and Frederickson, 2005; Georgiou et al., 2008a; Poulsen et al., 2015). Finally, although discrete naming (the ability to name stimuli presented one at-a-time) involves as much phonological processing as serial RAN (when all stimuli are presented in an array), it is less well correlated with reading than serial RAN (e.g., Georgiou et al., 2013; Logan and Schatschneider, 2014). This suggests that access to and retrieval of phonological representations from long-term memory is unlikely to be the reason (or the only reason) why RAN is related to reading.

Based on the finding that phonological awareness and RAN have additive effects on reading, Bowers and colleagues (e.g., Bowers and Wolf, 1993; Bowers et al., 1999; Sunseth and Bowers, 2002) proposed an alternative theoretical account according to which RAN predicts reading through the effects of orthographic processing. Orthographic processing occurs when groups of letters or entire words are processed as single units rather than as a sequence of grapheme-phoneme correspondences (e.g., Ehri, 1987). According to Bowers and Wolf (1993), if letter identification proceeds too slowly, as indexed by slow naming speed performance, letter representations in words will not be activated quickly enough to induce sensitivity to commonly occurring orthographic patterns. In support of this theoretical account, Manis et al. (2000) showed that RAN was a unique predictor of orthographic processing. In addition, Sunseth and Bowers (2002) showed that children with a naming speed deficit experience significant deficits in orthographic processing compared to children with no naming deficit. However, there is also evidence challenging this theoretical account. For example, some studies have reported weak or non-significant correlations between RAN and measures of orthographic processing (e.g., Compton et al., 2001; Conrad and Levy, 2007; Georgiou et al., 2008b). In addition, some studies have shown that RAN predicts reading over and above the effects of orthographic processing (e.g., Cutting and Denckla, 2001; Georgiou et al., 2008a; Liao et al., 2015).

Finally, some researchers have attributed the RAN-reading relationship to domain-general factors that affect performance in both RAN and reading. Kail and colleagues (e.g., Kail and Hall, 1994; Kail et al., 1999), for example, have argued that RAN and reading are related because skilled performance in both naming and reading depends, in part, on the speed with which the underlying processes are executed. Nicolson and colleagues (e.g., Nicolson and Fawcett, 1990; Nicolson et al., 2001), in turn, attributed the RAN-reading relationship to the function of the cerebellum. According to this theoretical account, cerebellar abnormality at birth leads to motor and articulatory problems, which, in turn, lead to slow naming speed and reading difficulties. More recently, Amtmann et al. (2007) proposed that RAN and reading are related because both require the maintenance of a set of names in working memory that allows the time-sensitive integration of phonological and orthographic representations of names. Finally, some researchers have suggested that attentional processes (e.g., response inhibition) may be responsible for the RAN-reading relationship (e.g., Semrud-Clikeman et al., 2000; Shao et al., 2013; Bexkens et al., 2015). RAN tasks usually involve naming 50 stimuli from a set of five different exemplars of a category (e.g., digits). This implies that these five stimuli are maintained in working memory in a highly accessible condition and that the activations of previously named stimuli compete with the current stimulus for response selection. Consequently, inhibition of inappropriate responses is necessary in order to select between competing alternatives. However, similar to the phonological and orthographic processing theoretical accounts, there is evidence showing that RAN is not strongly related to measures of speed of processing (e.g., Bowey et al., 2005; Georgiou et al., 2009b), motor programming (e.g., Raberger and Wimmer, 2003; Savage et al., 2007), working memory (e.g., Swanson and Kim, 2007; Georgiou et al., 2009a), or inhibition (e.g., Savage et al., 2007; Swanson et al., 2011; Altani et al., 2016).

The inability of the aforementioned theoretical accounts to explain the RAN-reading relationship in its totality could partly be attributed to the fact that the existing theoretical accounts have been examined mostly in isolation (see Cutting and Denckla, 2001; Georgiou et al., 2009b; Juul et al., 2014, for a few exceptions) and evidence has been sought to support or disprove specific explanations (e.g., Kail et al., 1999; Powell et al., 2007; Moll et al., 2009). The few studies that considered multiple pathways between RAN and reading have provided mixed findings (e.g., Cutting and Denckla, 2001; Holland et al., 2004; Juul et al., 2014; Georgiou et al., 2016). Cutting and Denckla (2001), for example, found that RAN, phonological awareness, and orthographic processing all had direct effects on reading and that RAN had no direct effects on either phonological awareness or orthographic processing. In contrast, Holland et al. (2004) found that the best fitting model was one in which RAN predicted reading indirectly through the effects of phonological awareness and orthographic processing. Finally, Georgiou et al. (2016) found that RAN predicted reading through phonological processing and orthographic processing only when speed of processing was not included in the model. The inclusion of speed of processing eliminated the relationship of RAN with phonological and orthographic processing. In addition, to date, no study has examined the direct effects of RAN on reading in conjunction with other underlying processes such as working memory, attention, and motor skills. Taken together, the findings of these studies suggest that RAN may be related to reading in more than one way and that the effect of specific mechanisms may depend on the inclusion or not of other mechanisms in the same model.

### The present study

**Figure 1:**
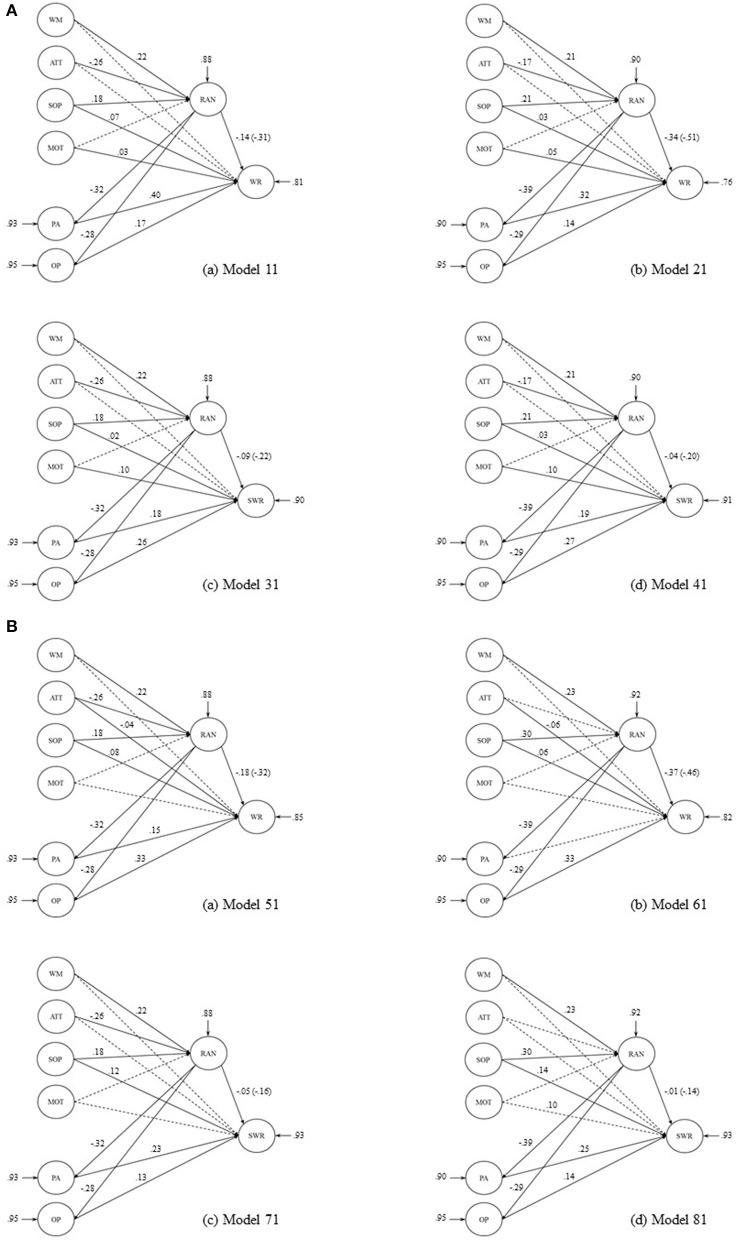
**(A)** The first set of models testing the direct and indirect effects of RAN, mediated by phonological ability and orthographic processing, to oral (M11, M21) and silent (M31, M41) reading fluency, in the concurrent analysis. The circles represent the latent cognitive (RAN, working memory, attention, processing speed), linguistic (phonological awareness and orthographic processing), motor skills, and reading (oral or silent word reading) variables. Coefficients are presented in standardized form. Total effects are given in parenthesis. Dotted lines indicate non-significant coefficients. Abbreviations for factors and variables: WM, working memory; ATT, attention; SOP, speed of processing; MOT, motor skills; PA, phonological ability; OP, orthographic processing; WR and SWR, oral and silent word reading, respectively. **(B)** The second set of models testing the direct and indirect effects of RAN, mediated by phonological ability and orthographic processing, to oral (M51, M61) and silent (M71, M81) reading fluency, in the longitudinal analysis. The circles represent the latent cognitive (RAN, working memory, attention, processing speed), linguistic (phonological ability and orthographic processing), motor skills, and reading (oral or silent word reading) variables. Coefficients are presented in standardized form. Total effects are given in parenthesis. Dotted lines indicate non-significant coefficients. Abbreviations for factors and variables: WM, working memory; ATT, attention; SOP, speed of processing; MOT, motor skills; PA, phonological awareness; OP, orthographic processing; WR and SWR, oral and silent word reading, respectively.

The purpose of this study was to examine the possible pathways between RAN and word reading fluency by simultaneously considering the prominent explanations of the RAN-reading relationship in a sample of Greek-speaking children followed from Grade 1 to Grade 2. The theoretical accounts discussed earlier were used to develop a model of word reading fluency (oral and silent) that incorporated direct and indirect relationships among the proposed variables (see Figures 1A,B).

The present study addresses five important gaps in the literature: First, to our knowledge, this is the first study to simultaneously contrast multiple rival theoretical accounts of the RAN-reading relationship. This allows us to obtain a more comprehensive picture of the processing skills that may underlie the RAN-reading relationship in accord with Wolf and Bowers' (1999) description of RAN. For example, as Georgiou et al. (2016) have shown, the inclusion or not of speed of processing in a model alters the relationship between RAN and orthographic processing. A similar finding may be expected in our study in relation to the role of motor skills since they have been linked to both phonological awareness and RAN (Nicolson et al., 2001), and phonological awareness and RAN are related (albeit modestly; Swanson et al., 2003). Second, we employed a longitudinal design^1^ following the same children from Grade 1 to Grade 2. This is particularly important in light of arguments that the role of different skills, such as phonological processing and orthographic processing, in the RAN-reading relationship may change across time (e.g., Bowey et al., 2005; Georgiou et al., 2008b). If the role of these processing skills in the RAN-reading relationship changes (e.g., the role of phonological processing decreasing across time and the role of orthographic processing increasing across time), then a longitudinal study is needed to capture these developmental changes. Third, the few studies that contrasted rival theoretical accounts of the RAN-reading relationship have recruited children from Grade 3 or higher (e.g., Cutting and Denckla, 2001; Holland et al., 2004; Powell et al., 2007; Georgiou et al., 2016; see Juul et al., 2014, for an exception). However, evidence suggests that RAN exerts a stronger effect on word reading when assessed in the early grades (e.g., Kirby et al., 2003; Araújo et al., 2015). Thus, we have the opportunity to test RAN's effects on reading at its best. Fourth, the naming model proposed by Wolf and Bowers (1999) pertained to letter naming. Given the findings of studies showing that non-alphanumeric RAN (Objects and Colors) is also a significant predictor of reading (e.g., Meyer et al., 1998; Kirby et al., 2003; Lervåg and Hulme, 2009; Araújo et al., 2015), it is worth investigating whether non-alphanumeric RAN is related to reading for the same reason(s) as alphanumeric RAN. Finally, we examined how RAN is related to both oral- and silent-reading fluency. This has implications for the role of articulation in the RAN-reading relationship. If articulation plays a significant role in the RAN-reading relationship, RAN should predict more strongly oral reading fluency than silent reading fluency (see Georgiou et al., 2013; van den Boer et al., 2014, for some preliminary evidence in support of this argument).

## Methods

### Participants

Two hundred eighty-six Greek-speaking children (143 males and 143 females) from Cyprus participated in the study. The mean age of the group in the initial assessment (Grade 1) was 6 years and 6 months (*SD* = 0.30 years) and in the follow-up assessment (Grade 2), 7 years and 5 months (*SD* = 0.31 years). Group's verbal (Similarities and Vocabulary; WISC-III-R; Wechsler, 1992) and non-verbal (Non-verbal Matrices; Cognitive Assessment System, CAS; Naglieri and Das, 1997) ability was assessed in Grade 1, all yielding average performance on the basis of normative scores in Greek (see Georgas et al., 1997; Papadopoulos et al., 2008a, for WISC-III-R and CAS norms, respectively). Almost half of the parents of the participating group were college or university graduates (46%) and the remaining were high school graduates (54%), consistent with the numbers provided by the Annual Survey of the Cyprus Statistical Service (2006; 45.3 and 54.7%, respectively). In regards to the community settings, approximately 62% of the sample was coming from urban communities and 38% from rural communities. These values are also in accord with the composition of the Greek-Cypriot population with 68.4% residing in urban settings and 31.6% residing in rural settings. Taken together, these data indicate that our sample was representative of children in the Greek-Cypriot population. School and parental consent for participation in the study was obtained prior to testing.

### Measures

#### Rapid automatized naming (RAN)

RAN was assessed with four measures: two alphanumeric (RAN-Digits and RAN-Letters) and two non-alphanumeric (RAN-Objects and RAN-Colors) tasks. All four measures included two cards (one relatively easy and one more difficult) that consisted of 20 testing items each (five different stimuli repeated four times each). The items in each task were presented in a semi-random order on a single page, with four lines of five items per page. Prior to testing, participants named the stimuli in a practice trial to ensure familiarity. In all tasks, participants' score was the ratio between the number of items named correctly and the time taken (correct/time), for each pair of tasks (averaged across the two cards). The naming errors were negligible (less than 1 per page) and for this reason they were not considered further. Test-retest reliability coefficients for a randomly-selected subsample of children in our study (*n* = 50) were as follows, for Grades 1 and 2, respectively: for RAN-Colors 0.83 and 0.80; for RAN-Objects 0.87 and 0.85; for RAN-Digits 0.85 and 0.89; and for RAN-Letters 0.79 and 0.82, respectively.

#### RAN-colors

Five basic and relatively more common colors, namely, */κόκκινo/πράσινo/κίτρινo/μπλε/άσπρo/; /kokkino/prasino/kitrino/mple/aspro/*; red, green, yellow, blue, and white were included in the first card. In contrast, the second card comprised of less common colors such as */ρoζ/γάλάζιo/κάφέ/πoρτoκάλί/μωβ/; /roz/galazio/kafe/portokali/mov/*; pink, light blue, brown, orange, and purple.

#### RAN-objects

This measure was modeled after the object naming task developed by Wimmer et al. (2000) and used initially by Papadopoulos et al. (2004). The words of the first card started with the same single consonant cluster (*κάπέλo/κάρέκλά/κεράσι/κάρότo/κλειδί; /kɑpɛlo/kɑrɛklɑ/kerɑsɪ/karoto/kliðɪ/*; hat, chair, cherry, carrot, key) whereas the words of the second card started with different consonant clusters (*φράoυλά/πλυντήριo/σκύλoς/στάυρός/μπάνάνά; /fraʊla/plintirio/skilos/staʌros/banana/*; strawberry, washing machine, dog, cross, banana).

#### RAN-digits

The digits from 1 to 5 were included in the first card. The second card comprised of digits 6–9 and 0 (zero).

#### RAN-letters

The letters of the first card were only vowels (ά, η, ɛ, o, υ); and the letters of the second card were only consonants, which are visually confusing (π, τ, σ, δ, θ).

#### Phonological awareness

Participants' phonological awareness skills were assessed with two tasks, namely Phoneme Elision and Phoneme Blending that have undergone extensive validation in previous studies (see Papadopoulos et al., 2009b, 2012). Both tasks were made up of 15 items. Prior to testing, participants did two sample items for practice in which feedback was provided. Both tasks were discontinued after four consecutive errors. A participant's score was the total number of correct responses in each task.

#### Phoneme elision

In this task, participants were asked to repeat a word after deleting an identified phoneme. The position of the phoneme to be deleted varied across items. After deleting the target phoneme, the remaining phonemes formed a word (e.g., *say* τώρά; /tora/; now, say /tora/ without saying the/t/sound 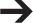 ώρά/ora/;time). Papadopoulos et al. (2012) reported Cronbach's alpha reliability coefficients to be 0.93 and 0.88 for Grades 1 and 2, respectively.

#### Phoneme blending

In this task, audio prompts presented the sounds of two-to-six sound words separately (*n* = 15), and participants were asked to orally blend them and say the resulting word. The words got progressively longer. The first four words consisted of two- to four- phoneme segments that followed the CV or CVC structure (e.g., φ*ως*; /fos/; light). The more difficult items contained more complex phoneme segments such as CCV (e.g., στόμά; /stoma/; mouth). Papadopoulos et al. (2012) reported Cronbach's alpha reliability coefficients to be 0.90 and 0.85 for Grades 1 and 2, respectively.

### Working memory

#### Word series

This task was adapted from Naglieri and Das (1997; see Papadopoulos et al., 2008a) and required participants to repeat a series of words in the same serial order they heard them from the examiner. The series increased in length from two to nine words (e.g., “*mama-gata-doro*”; *μάμά-γάτά-δώρo*). All of the nine words used were highly familiar and phonetically dissimilar. The number of word series recalled correctly was the participants' score (max = 27). Papadopoulos et al. (2008a) reported Cronbach's alpha reliability coefficient to be 0.77 and 0.83, for Grades 1 and 2, respectively.

#### Sentence questions

This task was also adapted from Naglieri and Das (1997; see Papadopoulos et al., 2008a) and required participants to answer questions about non-sensical sentences in which the content words had been replaced by color words (e.g., “*The yellow greened the blue*”; *τo κίτρινo πράσίνισɛ τo μπλɛ; “Who greened the blue?”; πoιoς πράσίνισɛ τo μπλɛ;*). Sentences started with 7 words and ended with 17 words. The participants' score was the number of correctly answered questions (max = 21). The task was discontinued after four consecutive errors. Papadopoulos et al. (2008a) reported Cronbach's alpha for this task to be 0.84 and 0.87 for Grades 1 and 2, respectively.

### Attention

#### Expressive attention

This task is based on the Stroop task, which has been widely used as a measure of interference and executive control (see McLeod, 1991; Nigg, 2001, for reviews). The version used in this study involved naming, as fast as possible, the color with which the words red, blue, yellow, and green, were printed instead of reading the words themselves. The index that was used in this test was the ratio score expressed as the time taken to complete the condition divided by the number of correct answers given by the participant. Papadopoulos et al. (2008a) reported Cronbach's alpha for this task to be 0.74 and 0.80 for Grades 1 and 2, respectively.

#### Selective attention

This task was also adapted from Naglieri and Das (1997; see Papadopoulos et al., 2008a). Participants were presented with a number of stimuli and they were asked to select a response by underlining numbers appearing in a particular form. One condition of this task was presented (Item 3 of the CAS battery) in which the targets were the digits “1,” “2,” and “3,” which had to be underlined only in the case they were presented in bold (as opposed to regular print). A ratio score expressed as the time elapsed, divided by the number of correct responses, was used as the participants' score. Papadopoulos et al. (2008a) reported Cronbach's alpha for this task to be 0.72 and 0.82 for Grades 1 and 2, respectively.

### Processing speed

#### Matching numbers

This task was also adapted from Naglieri and Das (1997; see Papadopoulos et al., 2008a). Participants were presented with two pages containing eight rows of numbers that were increasing in size and were instructed to underline the two numbers in each row that were the same (e.g., 18 22 25 17 33 22). The time and number correct for each page was recorded. To calculate the participants' score, we first divided the number correct in each page by the total time and then averaged the score in the two pages. Papadopoulos et al. (2008a) reported Cronbach's alpha for this task to be 0.79 and 0.81 for Grades 1 and 2, respectively.

#### Cancellation

In this task, participants were given two pages each consisting of 50 pairs of drawings and were asked to cancel (cross-out) those pairs that were identical in appearance (physical matching). The ratio score, expressed as the time taken divided by the number of correct responses, was used as the participant's processing speed score. Papadopoulos et al. (2008a) reported Cronbach's alpha for this task to be 0.70 and 0.69 for Grades 1 and 2, respectively.

### Motor skills

#### Bead threading

This task was adopted from the DST-J (Fawcett and Nicolson, 2004; see Papadopoulos et al., 2008b). Participants were given a basket of 15 round wooden beads and a cord and were asked to hold the cord in their “writing hand” and thread as many beads as possible in 30 s. The participants' score was the number of beads threaded in 30 s minus three beads threaded during practice. Test-retest reliability coefficients of Bead Threading were estimated using Cohen's kappa for a subsample of participants in our study (*n* = 50) as follows: 0.74 and 0.78 for Grades 1 and 2, respectively (*p*s < 0.01).

#### Postural stability

This task was also adopted from the DST-J (Fawcett and Nicolson, 2004; see Papadopoulos et al., 2008b). The task was delivered using a balance tester, a plastic device with a collar that slides on a cylindrically shaped shaft from one end to the pommel. A felt washer adjusted the collar to different resistance levels. The examiner stood behind the participant, placed the pommel end on the student's back, approximately two vertebrae above the waist. The collar was pushed along the length of the balance tester stopping before meeting the pommel, with a force of 2.5 Kg. The degree of “sway” displayed by the participant was rated using a six point scale for four trials (max = 24). For trials 1–2, the participant stood erect with arms at his/her side; for trials 3–4, the participant extended his/her arms in front at a 90° angle to the floor. The participants' score was the sum across the four trials. Test-retest reliability coefficients of Postural Stability were estimated using Cohen's kappa for a subsample of children in our study (*n* = 50) as follows: 0.81 and 0.82 for Grades 1 and 2, respectively (*p*s < 0.01).

### Orthographic processing

#### Orthographic choice

This task was adapted in Greek (Papadopoulos et al., 2009a) from the work of Olson and colleagues (e.g., Olson et al., 1989, 1994). It consisted of 20 items that were constructed in a way that phonological transcription alone did not reliably result in identifying the orthographically correct word among the three words included in each item (e.g., *άρέσɛι/άρέσι/άρέσoι;/ aresi/; like*). Participants were asked to select the correctly spelled word. The participants' score was the total number correct (max = 20). Cronbach's alpha reliability coefficient in the present sample was 0.68 in Grade 1 and 0.77 in Grade 2.

#### Two-minute spelling (TMS)

This task was also adopted from the DST-J (Fawcett and Nicolson, 2004; *Greek standardization*: Papadopoulos et al., 2008b) and was used to assess participants' orthographic processing skills. This task involves speed of writing as well as accuracy of the spelling. Participants were asked to spell a certain amount of words (up to 32 two- to multi-syllabic words) within 2 min. Cronbach's alpha for this task in our sample was 0.78 in Grade 1 and 0.80 in Grade 2.

#### Oral reading fluency

Two measures were used to assess participants' oral reading fluency, namely word reading fluency and a phoneme decoding fluency (Papadopoulos et al., 2008c). In both tasks, the reading speed (fluency) score, that is the number of words read correctly within 60 s, was recorded for each participant. We used fluency tasks because, by the end of Grade 1, Greek children reach ceiling on accuracy (almost 98% for real word reading and 92% for pseudoword reading; e.g., Papadopoulos, 2001; see also Seymour et al., 2003, for similar findings). Both the real word and the non-word lists were preceded by a practice list to familiarize participants with the task requirements.

#### Word reading fluency (WRF)

This test consists of 80 words forming a 2 × 2 × 2 factorial design in terms of frequency (high/low), orthographic regularity (regular/irregular; e.g., */τόπι/; /topi/; ball* vs. */έννoιά/; /ennia/*; concept), and length (bisyllabic/trisyllabic). The words were sampled from a large corpus of contemporary Greek and word frequency lists for ages 5 through 12 (Papadopoulos and Loizou, 2008). The words were mainly nouns with a few adjectives and verbs. Papadopoulos et al. (2008c) reported Cronbach's alpha for this task to be 0.97 and 0.81 in Grades 1 and 2, respectively.

#### Phonemic decoding fluency (PDF)

This task consists of 45 pronounceable non-words that are derived from real words (sampled also from the same corpus) after changing two or three letters (either by substituting them or using them backwards). The task started with bisyllabic words and ended with five-syllable words. Papadopoulos et al. (2008c) reported Cronbach's alpha for this task to be 0.92 in Grade 1 and 0.70 in Grade 2, respectively.

#### Silent reading fluency

Silent reading fluency was assessed with the Word Chains task, which has been adapted from the work of Jacobson and colleagues (e.g., Jacobson, 1999; Jacobson and Lundberg, 2000) and used in previous studies (e.g., Papadopoulos et al., 2009a; Georgiou et al., 2013). In this task, participants were asked to scan words presented as a continuous line of print without inter-word spaces (e.g., β*ιβλιoκάλo*φ*ως; bookgoodlight*). Participants were given 1 min and were asked to identify the words in each row by drawing a line to indicate where the spaces should be (e.g., *book/good/light*). The test included 15 rows of words of increasing length. The first two rows consisted of two words put together whereas the last three items consisted of seven words put together. The participants' score on this task was the number of correctly placed slashes. Papadopoulos et al. (2008c) reported Cronbach's alpha reliability to be 0.72 in Grade 1 and 0.83 in Grade 2.

### Procedure

Participants were individually tested in a quiet room in their schools between February and April (6–8 months after the beginning of the school year) by trained assistants. Testing lasted approximately 60 min in Grade 1 and 15 min in Grade 2. In Grade 1, participants were administered the RAN, working memory, attention, processing speed, motor skills, phonological awareness, orthographic processing, and reading fluency measures. In Grade 2, the participants were reassessed only on the reading fluency measures. The order of the tasks was similar for all participants within each grade.

### Statistical analysis

To examine the possible pathways between RAN and reading, we compared alternative models representing the prominent theoretical explanations of the RAN-reading relationship. Our aim was to select the best-fitting models explaining how RAN is related to both oral- and silent-reading fluency, concurrently (in Grade 1) and longitudinally (in Grade 2). We first evaluated the fit of a measurement model testing the intercorrelations and factor structure of the set of skills in Grade 1. Specifically, we conducted a confirmatory factor analysis examining the indicators of each latent factor and the intercorrelations between the latent factors. The model included seven factors: RAN, working memory, attention, processing speed, motor skills, phonological awareness, and orthographic processing. To evaluate the goodness of fit of the model to the data, we used a set of fit indexes: the chi-square value, the comparative fit index (CFI), and the root mean square error of approximation (RMSEA). Given the size of the present sample (*n* > 200), the ratio between chi-square and degrees of freedom was also used to judge model fit (Kline, 2011).

Second, we tested eight different models for each set of analyses: four of these models tested the effects of cognitive (RAN, working memory, attention, processing speed), linguistic (phonological awareness and orthographic processing), and motor skills on oral reading fluency, and the other four models tested the effects of cognitive, linguistic, and motor skills on silent reading fluency. In either case, two of the models included non-alphanumeric RAN measures as indicators of RAN and the other two models included alphanumeric RAN measures (digits and letters) as indicators of RAN. Also, in half of the models, we tested the direct effects of RAN to word reading fluency along with its indirect effects (Figures 1A,B). In the other half, only the indirect effects of RAN were included in the analysis. Finally, in all instances, and in accordance to the theoretical assumptions of the present study, attention, working memory, processing speed and motor skills acted as “common cause” variables predicting both RAN and reading fluency. The full models are displayed in the figures.

Each of the model testing the direct effects of RAN on word reading fluency (the *full* model) was compared to an alternative nested model (the *constrained* model) in which the direct effects of RAN on the dependent measure were fixed to zero (0). This constrained model tested the indirect effects of RAN on word reading against a full model that did not include the constraint. All nested models were directly compared using a chi-square difference test, which, in turn, allowed for the selection of the most parsimonious, best-fitting model.

## Results

### Preliminary analysis

First, we examined the distributional properties of all measures in both grades. We found no significant departures from normality (Tabachnick and Fidell, 2007). The means, standard deviations, and range of scores for all the variables are shown in Table 1. Next, we performed a correlational analysis between predictor (Grade 1) and criterion variables (Grades 1 and 2; see Table 2). As expected, the four RAN measures were strongly related to each other. Likewise, the measures of phonological awareness, working memory, attention, processing speed, motor skills, and orthographic processing were significantly interrelated. Importantly, all three word reading measures were significantly interrelated in both Grades (range for Grade 1: 0.32–0.75, and range for Grade 2: 0.27–0.69), with the relations between word reading fluency and silent word reading being modest in both Grades (0.32 and 0.38, for Grades 1 and 2, respectively). Also, the Word Reading Fluency and Phonemic Decoding Fluency tests were significantly related to all measures in both grades except from the measures of attention, processing speed, and motor skills (range for Grade 1: −0.14 to 0.14, and range for Grade 2: −0.06 to 0.10). Silent Reading Fluency, however, did not show the same consistency; although it correlated significantly to most of the measures, the correlations were relatively weak (range: −0.06 to 0.34 for Grade 1, and −0.04 to 0.33 for Grade 2).

**Table 1:** **Descriptive statistics on RAN, Phonological ability, Working Memory, Attention, Processing Speed RAN, Memory, Semantics, Reading, and Orthographic Measures**.

	**Grades**					
	**Grade 1**			**Grade 2**		
**Variables**	**M**	**(SD)**	**Range**	**M**	**(SD)**	**Range**
**RAN**						
Colors^a^	1.45	(0.45)	0.80–3.35			
Objects^a^	1.53	(0.38)	0.80–2.83			
Digits^a^	0.77	(0.20)	0.40–1.63			
Letters^a^	0.87	(0.27)	0.45–2.20			
**PHONOLOGICAL AWARENESS**						
Phoneme Elision	8.66	(4.97)	0–15			
Phoneme Blending	8.43	(4.33)	0–15			
**WORKING MEMORY**						
Word Series	7.88	(2.87)	0–17			
Sentence Questions	6.06	(2.92)	0–16			
**ATTENTION**						
Expressive Attention^a^	0.69	(0.16)	0.20–1.58			
Selective Attention^a^	0.10	(0.02)	0.02–0.18			
**PROCESSING SPEED**						
Matching Numbers^a^	0.08	(0.02)	0.02–0.17			
Cancellation^a^	0.18	(0.04)	0.02–0.30			
**MOTOR SKILLS**						
Bead Threading	7.01	(2.05)	2–12			
Postural Stability	1.90	(2.79)	0–16			
**ORTHOGRAPHIC PROCESSING**						
Orthographic Choice	8.53	(2.62)	0–14			
Two-Minute Spelling	4.25	(2.05)	0-11			
**ORAL READING FLUENCY**						
Word Reading Fluency	21.57	(8.55)	0–64	43.42	(10.97)	11–82
Phonemic Decoding Fluency	14.69	(5.59)	0–37	25.19	(6.07)	4–45
**SILENT READING FLUENCY**						
Word Chains	4.33	(2.83)	0–14	12.21	(4.40)	2–23

*N = 286 in both Grades*.

a *Ratio score (accuracy/time). Accuracy scores are reported for the remaining tasks. Word reading and Silent word reading scores were administered only in Grade 2 following the study aims. Values in the column Range are the empirical minimum and maximum scores. The possible maximum values for the accuracy scores are provided in the Method section in the description of the measures*.

**Table 2:** **Correlations Among all Measures in Grades 1 and 2**.

	**2**	**3**	**4**	**5**	**6**	**7**	**8**	**9**	**10**	**11**	**12**	**13**	**14**	**15**	**16**	**17**	**18**	**19**	**20**	**21**	**22**
1. RAN-C	0.55	0.51	0.42	−0.31	−0.30	−0.13	−0.21	0.37	0.19	0.26	0.22	−0.13	−0.05	−0.13	−0.25	−0.33	−0.32	−0.25	−0.31	−0.19	−0.21
2. RAN-P		0.55	0.47	−0.34	−0.32	−0.24	−0.31	0.38	0.26	0.31	0.26	−0.20	−0.09	−0.17	−0.25	−0.33	−0.24	−0.22	−0.38	−0.21	−0.20
3. RAN-D			0.68	−0.39	−0.38	−0.20	−0.21	0.35	0.23	0.31	0.25	−0.15	−0.16	−0.21	−0.28	−0.50	−0.40	−0.19	−0.45	−0.28	−0.21
4. RAN-L				−0.41	−0.37	−0.25	−0.25	0.32	0.18	0.26	0.23	−0.24	−0.10	−0.20	−0.23	−0.51	−0.47	−0.26	−0.43	−0.36	−0.21
5. PE					0.75	0.26	0.37	−0.15	−0.10	−0.20	−0.17	0.06	0.15	0.38	0.22	0.50	0.51	0.34	0.39	0.28	0.31
6. BL						0.23	0.38	−0.13	−0.07	−0.18	−0.15	0.01	0.14	0.40	0.15	0.48	0.44	0.28	0.38	0.22	0.33
7. WS							0.58	−0.15	−0.15	−0.18	−0.06	0.14	0.22	0.10	0.26	0.31	0.21	0.12	0.27	0.15	0.12
8. SQ								−0.15	−0.15	−0.21	−0.06	0.11	0.19	0.19	0.15	0.33	0.26	0.22	0.21	0.13	0.18
9. EA									0.50	0.35	0.27	−0.13	−0.11	−0.03	−0.06	−0.18	−0.19	−0.06	−0.10	−0.11	−0.04
10. SA										0.48	0.49	−0.05	−0.14	−0.07	−0.09	−0.18	−0.14	−0.10	−0.06	−0.11	−0.10
11. MN											0.47	−0.12	−0.13	−0.12	−0.11	−0.31	−0.23	−0.16	−0.28	−0.20	−0.25
12. CAN												−0.11	−0.10	−0.20	−0.07	−0.16	−0.08	−0.12	−0.11	−0.06	−0.13
13. BTHR													0.36	0.04	0.13	0.14	0.04	0.20	0.10	0.07	0.06
14. PSt														0.04	0.10	0.20	0.18	0.11	0.14	0.15	0.07
15. OC															0.33	0.36	0.26	0.32	0.26	0.18	0.21
16. TMS																0.25	0.23	0.25	0.48	0.36	0.21
17. WRF_1																	0.75	0.32	0.54	0.42	0.26
18. PDF_1																		0.33	0.45	0.35	0.28
19. SRF_1																			0.29	0.21	0.38
20. WRF_2																				0.69	0.38
21. PDF_2																					0.27
22. SRF_2																					

*N = 286 in both Grades. Correlations below 0.13 are non-significant, correlations between 0.13 and 0.19 are significant at the 0.05 level, and correlations higher than 0.19 are significant at the 0.01 level. RAN-C, Rapid Automatized Naming-Color; RAN-P, RAN-Pictures; RAN-D, RAN Digits; PE, Phoneme Elision; BL, Phoneme Blending; WS, Word Series; SQ, Sentence Questions; EA, Expressive Attention; SA, Selective Attention; MN, Matching Numbers; CAN, Cancellation; BTHR, Bead Threading; PSt, Postural Stability; OC, Orthographic Choice; TMS, Two-Minute Spelling; WRF, Word Reading Fluency; PDF, Phonemic Decoding Fluency; SWR, Silent Reading Fluency; x_1, indicates scores in Grade 1; x_2, indicates scores in Grade 2. Word reading and Silent word reading scores were administered only in Grade 2 following the study aims*.

### Results of structural equation modeling

The results of the measurement models indicated a good fit, for both non-alphanumeric [$\chi_{\left(58,\;N\;=\;286\right)}^{2}$ = 98.43, *p* < 0.001; CFI = 0.96; RMSEA = 0.05 (CI 0.90 = 0.03–0.06)] and alphanumeric models [$\chi_{\left(58,\;N\;=\;286\right)}^{2}$ = 110.77, *p* < 0.001; CFI = 0.95; RMSEA = 0.05 (CI 0.90 = 0.04–0.07)], suggesting that the postulated relationships in the models fitted the data relatively well.

Next, as shown in Table 3, the full models of oral reading fluency (Models 11, 21, 51, and 61) produced a χ^2^ that had a significantly better fit to the data than the constrained models, in both concurrent and longitudinal analyses (*p* < 0.05)^2^. The model indices indicated that the full models fitted the data well in both RAN non-alphanumeric, $\chi_{\left(12,\;\text{N}\;=\;286\right)}^{2}$ = 16.54, *p* < 0.05; CFI = 0.99; NFI = 0.96; and RMSEA = 0.04 (CI 0.90 = 0.00–0.07), and RAN alphanumeric, $\chi_{\left(12,\;\text{N}\;=\;286\right)}^{2}$ = 13.76, *p* < 0.001; CFI = 0.99; NFI = 0.97; and RMSEA = 0.02 (CI 0.90 = 0.00–0.07) in the concurrent analyses. Likewise, the model indices indicated that the full models fitted the data well in both RAN non-alphanumeric, $\chi_{\left(5,\;\text{N}\;=\;286\right)}^{2}$ = 5.13, *p* < 0.05; CFI = 0.99; NFI = 0.99; and RMSEA = 0.01 (CI 0.90 = 0.00–0.08), and RAN alphanumeric, $\chi_{\left(7,\;\text{N}\;=\;286\right)}^{2}$ = 12.54, *p* < 0.001; CFI = 0.98; NFI = 0.97; and RMSEA = 0.05 (CI 0.90 = 0.00–0.10) in the longitudinal analyses. It is noteworthy that no significant differences were observed between the nested models in any of the analyses performed with the silent reading fluency measure.

**Table 3:** **Fit Indices for Models of Concurrent and Longitudinal Analyses**.

**Model**	**χ^2^**	***df***	**CFI**	**NFI**	**RMSEA**	**90% CI**	**Predictors of reading measures**	**Predictors of RAN measures**	***R*^2^**	**Δχ^2^**
**CONCURRENT**										
M11: RAN_nal_-WR (dir)	16.54	12	0.99	0.96	0.04	0.00–0.07	RAN, OP, PA	ATT, WM, SOP	0.340	
M12: RAN_nal_-WR (indir)	22.61^*^	13	0.98	0.95	0.05	0.00–0.08	SOP, OP, PA	ATT, WM, SOP	0.323	6.07^*^
M21: RAN_al_-WR (dir)	13.76	12	0.99	0.97	0.02	0.00–0.07	RAN, OP, PA	ATT, WM, SOP	0.414	
M22: RAN_al_-WR (indir)	52.01^***^	13	0.91	0.89	0.10	0.07–0.13	SOP, OP, PA	ATT, WM, SOP	0.324	38.25^***^
M31: RAN_nal_-SWR (dir)	12.58	12	0.99	0.97	0.01	0.00–0.06	OP, PA	ATT, WM, SOP	0.185	
M32: RAN_nal_-SWR (indir)	14.86	13	0.99	0.96	0.02	0.00–0.06	OP, PA	ATT, WM, SOP	0.178	2.28
M41: RAN_al_-SWR (dir)	11.19	12	0.99	0.97	0.00	0.00–0.06	OP, PA	ATT, WM, SOP	0.179	
M42: RAN_al_-SWR (indir)	11.63	13	0.99	0.97	0.00	0.00–0.05	OP, PA	ATT, WM, SOP	0.178	0.44
**LONGITUDINAL^a^**										
M51: RAN_nal_-WR (dir)	5.13	5	0.99	0.99	0.01	0.00–0.08	RAN, OP, PA	ATT, WM, SOP	0.273	
M52: RAN_nal_-WR (indir)	14.18^*^	6	0.98	0.97	0.07	0.02–0.12	OP, PA	ATT, WM, SOP	0.249	9.05^**^
M61: RAN_al_-WR (dir)	12.54	7	0.98	0.97	0.05	0.00–0.10	RAN, OP	WM, SOP	0.333	
M62: RAN_al_-WR (indir)	54.61^***^	8	0.88	0.87	0.14	0.10–0.18	SOP, OP	WM, SOP	0.219	42.07^***^
M71: RAN_nal_-SWR (dir)	6.80	6	0.99	0.98	0.02	0.00–0.08	SOP, OP, PA	ATT, WM, SOP	0.138	
M72: RAN_nal_-SWR (indir)	7.42	7	0.99	0.98	0.02	0.00–0.07	SOP, OP, PA	ATT, WM, SOP	0.135	0.62
M81: RAN_al_-SWR (dir)	11.87	7	0.98	0.97	0.05	0.00–0.10	SOP, OP, PA	WM, SOP	0.136	
M82: RAN_al_-SWR (indir)	11.87	8	0.99	0.97	0.04	0.00–0.09	SOP, OP, PA	WM, SOP	0.136	0.01

N = 286; M, Model; Concurrent, Grade 1 RAN and cognitive scores predict Grade 1 reading scores; Longitudinal, Grade 1 RAN and cognitive scores predict Grade 2 reading scores; Mean age Wave 1, 5.8 (SD = 0.31); Wave 2 Mean age, 6.6 (SD = 0.31); Chi-square difference tests are comparisons to the (X)1-model; CFI, Comparative Fit Index; NFI, Normed Fit Index; RMSEA, Root-Mean-Square Error of Approximation; CI, Confidence Interval; parameters, parameters predicting dependent variable; WR, Word Reading; SWR, Silent Word Reading; PA, Phonological Awareness; OP, Orthographic Processing; SOP, Speed of Processing; dir, model with RAN direct effect on the dependent variable; indir, model without RAN direct effect on the dependent variable;

a Differences in dfs in the longitudinal analysis result from Wald test evaluations;

* p < 0.05;

** p < 0.01;

*** *p < 0.001*.

Moreover, a careful look at the predictors of reading fluency (in Table 3) suggests that orthographic processing, phonological awareness, and processing speed, along with RAN (with the latter observed only when oral reading fluency was the dependent variable) accounted for the largest portion of variance in reading fluency in both concurrent and longitudinal analyses. Also, with the exception of the silent reading fluency models in the longitudinal analyses, attention, working memory, and processing speed were found to be significant predictors of RAN performance.

Furthermore, we estimated the mediated effects in those models for which a significant change in χ^2^ was observed. Our aim was to examine which of the involved variables explained most of the variance in reading in order to better identify and explain the mechanism that underlies the relationship between RAN and reading. Table 4 presents the total, direct, and indirect effects and their standard errors of RAN on oral reading fluency. All effects were significant at the 0.05 level. As indicated by the estimates, the total effects of RAN—including the indirect effects of attention, working memory, and processing speed, the indirect effects of RAN through phonological awareness and orthographic processing, and the direct effects of RAN on reading—explained most of the variance in reading in the concurrent analysis, using both non-alphanumeric RAN (ĉ = −0.362, *s*_ĉ_ = 0.061, *t*_ĉ_ = 5.93) and alphanumeric RAN measures (ĉ = −0.551, *s*_ĉ_ = 0.054, *t*_ĉ_ = 10.16), compared to the direct effects of phonological awareness (ĉ = −0.399, *s*_ĉ_ = 0.054, *t*_ĉ_ = 7.36) and RAN only (ĉ = −0.359, *s*_ĉ_ = 0.056, *t*_ĉ_ = 6.39), respectively. Likewise, in the longitudinal analysis, the total effects of alphanumeric RAN measures (ĉ = −0.478, *s*_ĉ_ = 0.056, *t*_ĉ_ = 8.71) explained most of the variance in reading fluency with the direct effects of orthographic processing (ĉ = −0.400, *s*_ĉ_ = 0.062, *t*_ĉ_ = 6.41) following in size. Finally, the total direct effects of orthographic processing explained most of the variance in reading performance when non-alphanumeric RAN measures were included in the analysis (ĉ = −0.401, *s*_ĉ_ = 0.068, *t*_ĉ_ = 5.90), with the total effects of RAN (ĉ = −0.400, *s*_ĉ_ = 0.062, *t*_ĉ_ = 6.41) coming second. In general, these results indicate that the total effect that is equal to the sum of the direct effects of RAN and the indirect effects of attention, working memory, processing, phonological awareness, and orthographic processing better explains the relationship between RAN and reading fluency compared to the direct effects of RAN. Also, in Grade 1, phonological awareness was found to be an independent variable mediating the relationship between RAN and reading fluency. Similarly, in the longitudinal analysis, orthographic processing was found to be an independent variable mediating the RAN-reading relationship.

**Table 4:** **Total, Direct, and Indirect Effects of RAN on oral reading fluency**.

	**Oral reading fluency**			
**Effects**	**Estimate^a^**	**Standard Error^a^**	**Estimate^b^**	**Standard Error^b^**
**CONCURRENT**				
Total effects of RAN	−0.362	0.061	−0.551	0.054
Direct effects of RAN	−0.147	0.059	−0.359	0.056
Total indirect effects of RAN	−0.213	0.036	−0.191	0.032
Direct effect of PA	0.399	0.054	0.316	0.053
Direct effect of OP	0.202	0.066	0.168	0.062
Indirect effect of ATT	0.094	0.028	0.097	0.037
Indirect effect of WM	0.075	0.022	0.119	0.031
Indirect effect of SOP	0.066	0.025	0.118	0.038
**LONGITUDINAL**				
Total effects of RAN	−0.338	0.063	−0.478	0.056
Direct effects of RAN	−0.193	0.063	−0.380	0.056
Total indirect effects of RAN	−0.145	0.032	−0.098	0.025
Direct effect of PA	0.142	0.056	−	−
Direct effect of OP	0.401	0.068	0.400	0.062
Indirect effect of ATT	0.093	0.028	−	−
Indirect effect of WM	0.076	0.023	0.112	0.030
Indirect effect of SOP	0.066	0.026	0.148	0.033

a Analyses involving non-alphanumeric RAN measures;

b *Analyses involving alphanumeric RAN measures; PA, Phonological Awareness; OP, Orthographic Processing; ATT, Attention; WM, Working Memory; SOP, Speed of Processing. All effects are significant at the 0.05 level*.

Finally, to test further the hypothesis of longitudinal stability of the models, we fitted two multi-group models^3^ to the data comparing the most parsimonious models and explored whether the coefficients of the alphanumeric (M11–M51) and non-alphanumeric (M21–M61) models are invariant across the two grades. The results confirmed the existence of longitudinal stability of the models across grades [$\Delta \chi_{\left(7\right)}^{2}$ = 9.47, *p* = 0.22 and $\Delta \chi_{\left(8\right)}^{2}$ = 5.36, *p* = 0.71, for alphanumeric and non-alphanumeric models, respectively].

To summarize, the relationship between RAN and reading fluency is not explained only by the effects that RAN exerts on reading fluency, but also by the effects that phonological awareness, orthographic processing, attention, working memory and processing speed exert on reading fluency, with the former two, in particular, serving as mediators of the RAN-reading relationship.

## Discussion

The primary aim of the present study was to examine the role of RAN in word reading fluency by simultaneously contrasting the most prominent theoretical accounts of the RAN-reading relationship, in the early years of reading development. In what was found to be the best fitting model, RAN exerted direct effects on reading fluency, a result that was observed though only when oral reading fluency was the outcome measure. This was true for both non-alphanumeric and alphanumeric RAN measures, in both concurrent (Grade 1) and longitudinal (from Grade 1 to Grade 2) analyses, a finding that was further confirmed through longitudinal invariance testing.

These findings contribute to the existing literature in four important ways. First, we have shown that RAN is a unique predictor of oral reading fluency, but not of silent reading fluency. These results reinforce those of previous studies showing that RAN is more strongly related to oral reading fluency than silent reading fluency (e.g., Georgiou et al., 2013; van den Boer et al., 2014) and suggest that articulation is important for the RAN-reading relationship. Second, non-alphanumeric RAN tasks appear to predict oral reading fluency equally well as alphanumeric RAN tasks, and almost for the same reasons. The only difference was that the relationship between RAN and oral reading fluency was better explained by the direct effects of orthographic processing on reading along with those of RAN, when the non-alphanumeric RAN tasks were used in the longitudinal analysis. Thus, our findings provide additional evidence for the indistinguishable processing of different RAN stimuli in the early years and their significant relationship with word reading. In fact, van den Bos et al. (2002) have reported similar findings showing that all three measures, namely letters, numbers, and objects, obtained for a group of 8-year-olds in their study, were loading on the same factor and predicted reading equally well.

Third, in the models predicting oral reading fluency in which the direct effects of RAN were also included, phonological awareness and orthographic processing emerged as significant concurrent predictors of reading performance, a result to suggest that the effects of RAN on reading fluency are both direct and indirect (through phonological awareness and orthographic processing). In fact, as shown in Table 4, the predictive variance of RAN in reading fluency was found to be larger when this was mediated by phonological awareness (concurrently) and orthographic processing (longitudinally), rather than when RAN's effects were estimated separately on reading fluency. Furthermore, when the direct effects of RAN were excluded from the analyses, processing speed also emerged as a significant predictor of oral reading fluency. This finding is in line with the argument put forward by several researchers that processing speed partly mediates the RAN-reading relationship (e.g., Bowey et al., 2005; Georgiou et al., 2009b, 2012; Liao et al., 2015).

Fourth, in the models predicting silent reading fluency concurrently, phonological awareness and orthographic processing were found to be significant predictors of reading performance with processing speed sharing additional variance in the longitudinal analysis. This may partly reflect that when children are still learning to read and access to the phonological and orthographic codes has not yet become automatic, they tend to rely on the accurate serial processing of symbols. However, as automaticity in lexical access is achieved, skilled readers process more than one symbol at a time, making processing speed an equally significant predictor of reading (e.g., Protopapas et al., 2013; Georgiou et al., 2014; van den Boer and de Jong, 2015). These findings contribute new knowledge with respect to the importance of different skills in the RAN-reading relationship across time, when oral and silent word reading are assessed.

Perhaps the most interesting finding is the role working memory, attention, and processing speed play as *distal* “common cause” processes to the RAN-reading relationship. A closer look at the predictors of RAN (Table 3) and the estimates of the effects on reading (Table 4) reveals some interesting patterns of relationships. First, RAN seems to be consistently related to processing speed. It has been argued that RAN may be a manifestation of general processing speed, which is the speed at which cognitive processing occurs (e.g., Kail et al., 1999) and for this reason skilled performance in both naming and reading depends, in part, on the speed underlying processes are executed. This finding seems to be particularly true in languages with a transparent orthography in which reading fluency is often the key outcome measure (e.g., Papadopoulos et al., 2009a; Torppa et al., 2013). Our findings suggest that the reason why RAN predicts reading fluency is partly because speed is an integral part of RAN performance, and thus, it acts as a common cause variable in the RAN-reading relationship.

Similarly, the assumption that working memory plays a significant role in the RAN-reading relationship finds support in the present study. In fact, in addition to previous studies that have investigated and supported the direct predictive role of working memory in children's word reading (e.g., Jacobson et al., 2011) or reading comprehension (e.g., Leong et al., 2008; Kendeou et al., 2012; Weng et al., 2016), the present findings suggest that working memory may also be a common cause variable in the RAN-reading relationship. This is an expected result given the effortful nature of cognitive control required to successfully perform reading fluency tasks. Fluent reading activates a stored neural model which, in turn, allows not only fast reading to occur, but also activates correct pronunciation and understanding of the word (Daneman and Carpenter, 1980). These steps of controlled processing highlight the role of working memory in RAN and reading.

Our findings also suggest that response inhibition, as an index of attention, may partly explain why RAN is related to reading fluency, particularly in the earlier stages of reading (e.g., Shao et al., 2013; Bexkens et al., 2015). It is already known that RAN and reading are related because both require serial processing (Georgiou et al., 2013). However, for this serial processing to occur successfully, attention has to be disengaged from naming a current item and directed to the next item (Altani et al., 2016). In fact, recent studies using eye-tracking methodology have elucidated the influential role of attention on RAN performance (e.g., Jones et al., 2008, 2009; Kuperman et al., 2016). However, our results show that, in the early stages of reading, attention remains important, even when other concurrent predictors of the RAN-reading relationship are taken into account.

These results also suggest that a possible reason for the failure of previous studies to detect the consistent effects of working memory, attention, or processing speed on reading when these are examined in conjunction with the effects of RAN (e.g., Bowey et al., 2005; Georgiou et al., 2009b; Swanson et al., 2011) may be the fact that they have overlooked the indirect effects that this set of skills has on reading via RAN. These findings point to a more complex relationship among basic cognitive processing skills, RAN and reading, which warrants further investigation.

In addition, we found that phonological awareness and orthographic processing mediate the RAN-reading relationship. Although, previous research has consistently supported that phonological awareness (e.g., Bowey et al., 2005; Poulsen et al., 2015) and orthographic processing (e.g., Sunseth and Bowers, 2002; Georgiou et al., 2009b) play an important role in the RAN-reading relationship, less emphasis has been put on possible developmental changes in the role of these processing skills in the RAN-reading relationship. Our results showed that phonological awareness was a stronger mediator of the RAN-reading relationship in the concurrent analyses, whilst orthographic processing was a stronger mediator in the longitudinal analyses. This finding could be attributed in part to the nature of the shared components that these two skills have with RAN and in part to the transparency of the Greek orthography. While the role of phonological awareness and orthographic processing in reading across languages is still controversial (see Georgiou et al., 2008a; Vaessen et al., 2010), the role these two skills play in languages with a transparent orthography is clearer (e.g., Torppa et al., 2007; Landerl and Wimmer, 2008; Papadopoulos et al., 2009a). On the one hand, the transparency of the Greek language allows young readers to use the phonological representations of any grain-size units (rhyme, syllable, or phoneme) that are available to them (Papadopoulos et al., 2012). This possibility enables even children who show insufficient phonological processing at school entry to gradually tackle their difficulties with phonological processing and find means to compensate for their poor reading performance (Papadopoulos et al., 2009a). As a result, the contribution of phonological awareness to reading is time limited, and the present results indicate that this is also true for the mediating role of phonological awareness in the RAN-reading relationship. Di Filippo et al. (2005) have also reported similar findings from Italian showing the independent role of phonological awareness and RAN in predicting reading, with phonological awareness having time-limited effects on reading only among young children. On the other hand, although orthographic processing correlated concurrently with phonological awareness in Grades 1 and 2, it turned out to be a stronger mediator of the RAN-reading relationship in Grade 2. This finding, of course, has important educational implications, especially for children learning to read in a transparent orthography, as it shows that a solid foundation of phonological skills may be required for RAN skills to facilitate reading and before the complexity of the orthographic system can be fully processed.

Overall, the most important finding of the present study suggests that the direct effects of RAN alone may be less important for the prediction of oral reading fluency (at least in the earliest stages of learning to read), in spite of the emphasis placed on RAN in the relevant research. Another important finding is that among the prominent explanations of the RAN-reading relationship which were examined simultaneously, phonological awareness and orthographic processing were found to play a dominant role in this relationship. RAN was found to have a direct effect on reading (in partial agreement with the model proposed by Cutting and Denckla, 2001), but also to predict reading indirectly through the effects of phonological awareness and orthographic processing (in agreement with the model proposed by Holland et al., 2004). In addition, phonological awareness was found to contribute more to the RAN-reading relationship in the earlier phases of reading than later. At the same time, orthographic processing contributed more to the RAN-reading as children grew older. These results extend previous findings with older children (Grade 4) learning to read in Greek that have shown that RAN is a unique predictor of reading fluency and its effects are partly mediated by orthographic processing, particularly when operationalized with speeded measures (Georgiou et al., 2016). This developmental change may reflect the progress of reading itself from serial to a more holistic processing (see de Jong, 2011; Protopapas et al., 2013, for similar arguments using a different approach).

Finally, the present findings indicate that RAN performance is related to several processes measured with different neuropsychological measures, and that, although simple at the surface level, RAN tasks are multi-componential (Wolf et al., 2000). At the very least, it was confirmed that phonological and naming-speed factors may also exert independent effects on reading, which indicates that naming speed is distinct enough from phonological awareness to make a unique contribution in the prediction of word reading (Wolf and Bowers, 1999; Papadopoulos et al., 2009a). We believe that future studies need to address the same issues and explore systematically and longitudinally the prominent theoretical explanations of the RAN-reading relationship with cohorts of children exhibiting reading difficulties, using perhaps different methodological approaches and analyses, such as latent class regression modeling (e.g., Ozernov-Palchik et al., in press) or non-linear modeling, such as the cusp-catastrophe model (e.g., Sideridis et al., 2016).

Some limitations of the present study are worth mentioning. First, our study was conducted in Greek and therefore our findings may generalize only to orthographies that are similarly transparent (e.g., German, Spanish). Second, because of the transparency of the Greek language we could not administer any reading accuracy measures. Given that a connection between RAN and reading accuracy has been established in several languages (e.g., Swanson et al., 2003; Araújo et al., 2015), our findings should be replicated using also reading accuracy measures. Third, we assessed different processing skills that may explain the RAN-reading relationship only in Grade 1. If the role of different processing skills in the RAN-reading relationship changes across time (e.g., Georgiou et al., 2009b), then our study can only reveal what skills were important at the beginning of learning to read. Finally, our study was correlational in nature and the effects of the different variables on reading do not imply causation.

To conclude, this study has offered a different angle to an ongoing discussion about the nature of RAN and its relationship to reading. Perhaps the main question should no longer be whether RAN predicts reading fluency, accounting for a significant amount of variance above and beyond the effects of other cognitive or linguistic skills. Neither should it focus on the mediating role that other cognitive and linguistic processes may play in the RAN-reading relationship. Rather, the future of the research on RAN performance ought to lie in its ability to better define the properties of other processes (including those of reading; see de Jong, 2011) that RAN carries and how these are critical in determining naming speed's influential role on reading performance.

## Author contributions

Each author has participated sufficiently in the work to take responsibility for certain portions of the manuscript's content. TP made substantial contributions to conception and design, data collection, and analysis and interpretation of the data. GS made significant contributions to data analysis and interpretation of the data. GG made considerable contribution to conception and design and interpretation of the data.

## Funding

This research was supported by a Cyprus Research Promotion Foundation grant: NEA YΠOΔOMH/ΣTPATH/0308/37.

### Conflict of interest statement

The authors declare that the research was conducted in the absence of any commercial or financial relationships that could be construed as a potential conflict of interest.

## Footnotes

^1^We use the term *longitudinal* to refer to variables in Grade 1 predicting reading in Grade 2 and not to predictors assessed in different time points.

^2^Working memory was allowed to covariate with phonological awareness and orthographic processing in order to improve the fit of the models.

^3^Only the coefficients which were similar across the models were included in this set of analyses.
